# Bidirectional Associations Between Depressive and Anxiety Symptoms and Loneliness During the COVID-19 Pandemic: Dynamic Panel Models With Fixed Effects

**DOI:** 10.3389/fpsyt.2021.738892

**Published:** 2021-12-09

**Authors:** Cillian P. McDowell, Jacob D. Meyer, Daniel W. Russell, Cassandra Sue Brower, Jeni Lansing, Matthew P. Herring

**Affiliations:** ^1^The Irish Longitudinal Study of Ageing, Trinity College Dublin, The University of Dublin, Dublin, Ireland; ^2^School of Medicine, Trinity College Dublin, The University of Dublin, Dublin, Ireland; ^3^Department of Kinesiology, Iowa State University, Ames, IA, United States; ^4^Department of Human Development and Family Studies, Iowa State University, Ames, IA, United States; ^5^Physical Activity for Health Research Cluster, Health Research Institute, University of Limerick, Limerick, Ireland; ^6^Department of Physical Education and Sport Sciences, University of Limerick, Limerick, Ireland

**Keywords:** mental health, COVID-19, panel data, coronavirus, loneliness

## Abstract

**Background:** Understanding the direction and magnitude of mental health-loneliness associations across time is important to understand how best to prevent and treat mental health and loneliness. This study used weekly data collected over 8 weeks throughout the COVID-19 pandemic to expand previous findings and using dynamic panel models with fixed effects which account for all time-invariant confounding and reverse causation.

**Methods:** Prospective data on a convenience and snowball sample from all 50 US states and the District of Colombia (*n* = 2,361 with ≥2 responses, 63.8% female; 76% retention rate) were collected weekly *via* online survey at nine consecutive timepoints (April 3–June 3, 2020). Anxiety and depressive symptoms and loneliness were assessed at each timepoint and participants reported the COVID-19 containment strategies they were following. Dynamic panel models with fixed effects examined bidirectional associations between anxiety and depressive symptoms and loneliness, and associations of COVID-19 containment strategies with these outcomes.

**Results:** Depressive symptoms were associated with small increases in both anxiety symptoms (β = 0.065, 95% CI = 0.022–0.109; *p* = 0.004) and loneliness (β = 0.019, 0.008–0.030; *p* = 0.001) at the subsequent timepoint. Anxiety symptoms were associated with a small subsequent increase in loneliness (β = 0.014, 0.003–0.025; *p* = 0.015) but not depressive symptoms (β = 0.025, −0.020–0.070; *p* = 0.281). Loneliness was strongly associated with subsequent increases in both depressive (β = 0.309, 0.159–0.459; *p* < 0.001) and anxiety (β = 0.301, 0.165–0.436; *p* < 0.001) symptoms. Compared to social distancing, adhering to stay-at-home orders or quarantining were not associated with anxiety and depressive symptoms or loneliness (both *p* ≥ 0.095).

**Conclusions:** High loneliness may be a key risk factor for the development of future anxiety or depressive symptoms, underscoring the need to combat or prevent loneliness both throughout and beyond the COVID-19 pandemic. COVID-19 containment strategies were not associated with mental health, indicating that other factors may explain previous reports of mental health deterioration throughout the pandemic.

## Introduction

The COVID-19 pandemic and containment strategies employed to limit its spread (1–3) have profoundly impacted daily life in the US and globally, including substantial negative changes to health behaviors, employment, and mental health (4–8). Experts have expressed particular concern regarding potential increases in loneliness [or perceived social isolation; (9, 10)], defined as subjective distress resulting from a discrepancy between desired and perceived social relationships (11). Mortality attributable to low social support (162,000 deaths in 2000) exceeds that for cancer or stroke in the US (12), and in the UK it is estimated that the cost of loneliness to employers is more than $3 billion annually (13). Loneliness also has a substantial personal burden and is a major risk factor for physiological and health outcomes (14), including coronary heart disease and stroke, increased healthcare use in older people, cognitive decline, depression, and increased risk of all-cause mortality (15–19).

Loneliness and mental health are likely interrelated, underpinned by a combination of psychological and physiological pathways (20). Indeed, much research has examined the relationship between loneliness and depression, but many of these studies have important limitations (i.e., cross-sectional, small samples, and single-item measures of loneliness) and few have assessed bidirectional relationships (17, 21). Some evidence supports a reciprocal relationship (14, 22, 23), but research has not always been consistent (24). Cross-sectional evidence supports associations between loneliness and diagnosed anxiety disorders (25), although few studies have examined the bidirectional relationship between loneliness and anxiety. One such study demonstrated a bidirectional relationship between loneliness and social anxiety (26), while experimental evidence in which feelings of loneliness and social connectedness were hypnotically induced showed that loneliness increased anxiety (and depressive) symptoms (27). This is particularly concerning given the COVID-related deleterious impact on mental health (7), potentially creating a negative feedback loop between deteriorating mental health and loneliness. However, despite initial concerns and cross-sectional evidence of high pandemic-related levels of loneliness (9, 10), early longitudinal evidence suggested only minimal changes in loneliness (28). Nonetheless, loneliness also was not improved across time, and there will likely be longer-term effects of the pandemic; for example, living in economically and socially challenging conditions is associated with higher levels of loneliness (29).

Previous research examining longitudinal bidirectional relationships between depressive symptoms and loneliness across periods of years may not be generalizable to the rapid pandemic-related timeframes (i.e., weeks and months). Moreover, potential confounding from time-invariant factors (e.g., genetic susceptibility to loneliness and/or impaired mental health) and the time-varying effects of psychosocial risk factors known to be associated with both loneliness and depressive symptoms [e.g., low social network size and high perceived stress; (22, 24)] have not been adequately considered. It therefore remains plausible that the association between loneliness and depressive symptoms is, at least in part, attributable to these external factors. Additionally, research exploring potential bidirectional relationships between loneliness and anxiety symptoms is scarce.

Therefore, using dynamic panel models with fixed effects, the study reported here assessed: (1) longitudinal bidirectional relationships between anxiety and depressive symptoms and loneliness, and, (2) whether these associations might be attributable to perceived stress and social network size. These panel models address two central threats to valid causal inference in epidemiological studies, namely time-invariant confounding (e.g., genetics, sex, race, adverse childhood experiences, etc.) and reverse causation (30, 31).

## Methods

### Sample

This longitudinal study includes follow-up data from the *COVID-19 and Well-being Study* collected at Iowa State University, following approval as an exempt study by the University Institutional Review Board (IRB# 20-144-00). Data from this study have been utilized in previous publications (6, 32–36). Recruitment methods for the initial survey included: mass emails to Iowa State University students, faculty, staff, and alumni; snowball sampling; and posts to social media pages. Mass emails and posts included a link to an anonymous electronic survey for interested participants to read and consent to enrolment in the study and verify inclusion criteria of being ≥18 years of age and current US residence.

The initial survey took 20–30 min and was completed by 3,133 adults from all 50 US states and the District of Colombia from April 3^rd^-10th, 2020 who indicated interest in continued participation. Participants had the opportunity to provide consent to be re-contacted to complete 8 weekly abbreviated follow-up surveys. Follow-up surveys were sent every 7 days from initial survey completion for 8 weeks. In the current study, 772 adults who did not complete at least one follow-up survey were excluded, leaving a final sample size of 2,361.

### Measures

The 21-item Beck Depression Inventory-II (BDI-II), excluding the suicidality item, assessed depressive symptoms (37). Response options differed for each item but, for example, item 1 response options were “I do not feel sad” (scored as 0), “I feel sad much of the time” (scored as 1), “I am sad all the time” (scored as 2), and “I am so sad or unhappy that I can't stand it” (scored as 3). Scores were divided by 20 and multiplied by 21 to calculate estimated total scores ranging from 0 to 63, with higher scores indicating more depressive symptoms. The BDI-II has previously demonstrated internal consistency around α = 0.90 and test-retest reliability between *r* = 0.73–0.96 (38).

The 21-item Beck Anxiety Inventory (BAI) assessed anxiety symptoms (39). Response options for each item were “Not at all” (scored as 0), “Mildly, but it didn't bother me much” (scored as 1), “Moderately—it wasn't pleasant at times” (scored as 2), and “Severely—it bothered me a lot” (scored as 3). Thus, scores range from 0 to 63 with higher scores indicating more anxiety symptoms. The BAI has previously demonstrated internal consistency of α = 0.91 and test–retest reliability of *r* = 0.65 (40).

The 3-item Loneliness scale examined loneliness symptoms (41). This measure avoids use of the term “lonely” or “loneliness” and thus avoids much of the stigma associated with, and consequent underestimation of, loneliness. Response options for each item were “Hardly ever or never” (scored as 1), “Some of the time” (scored as 2), and “Often” (scored as 3). Thus, scores range from 3 to 9 with higher scores indicating more loneliness symptoms. It has previously demonstrated an internal consistency of α = 0.72 and correlation of *r* = 0.82 with the revised UCLA Loneliness Scale (41).

Social network size was assessed using an abbreviated version of the Lubben Social Network Scale-6 (42) with three items combining friends/relatives in each item. Questions assessed how many relatives/friends the respondent (1) speaks to at least once a day, (2) feels at ease with that they could talk about private matters, and (3) feels close to such that they could call on them for help. Response options were “None” (scored as 0), “One” (scored as 1), “Two” (scored as 2), “Three or four” (scored as 3), “Five through eight” (scored as 4), and “Nine or more” (scored as 5). Thus, scores range from 0 to 15 with higher scores indicating greater social network size. It has previously demonstrated internal consistency of α = 0.83 (42).

The 4-item Perceived Stress Scale-4 assessed stress. Response options for each item were “Never” (scored as 0), “Almost never” (scored as 1), “Sometimes” (scored as 2), “Fairly often” (scored as 3), and “Often” (scored as 4). Thus, scores range from 0 to 16 with higher scores indicating more perceived levels of stress. It has previously demonstrated internal consistency ranging between α = 0.60–0.82 (43).

Participants also indicated the COVID-19 containment strategies to which they were adhering (as opposed to those that were recommended in their area). Possible responses were:

1. Self-Isolation: For people who actually have the virus or suspect they may be infected. People who have been infected with the virus may be asked to self-isolate at home if they have no symptoms or are only mildly ill.
2. Quarantine: For those who may have been exposed to the virus. They are asked to stay at home. Some people may choose to be asked to self-quarantine, meaning they do it voluntarily because they think they may have been exposed or they are being cautious.
3. Shelter-in-place: People that are being asked to stay at home as much as possible, meaning they shouldn't be out unless getting food, gas, or other essentials, or for medical reasons.
4. Stay-at-home order: Residents can still go out for essential needs as long as they are practicing social distancing and “common sense.”
5. Social distancing: Means remaining out of congregate settings, avoiding mass gatherings, and maintaining distance (~6 feet or 2 m) from others when possible.

Participants selected all that applied and were grouped based upon the most restrictive strategy that they were following, with quarantine and self-isolation the most restrictive, shelter-in-place or stay-at-home next, and social distancing or none the least restrictive.

### Statistical Analyses

Analyses were conducted in STATA 14.2. Summary statistics were means and standard deviations for continuous variables and frequencies for categorical variables. *T*-tests and Cohen's *d* effect sizes assessed differences in anxiety and depressive symptoms and loneliness between participants with and without data at follow-up. Using the maximum likelihood–structural equation models method, dynamic panel models with fixed effects were applied to assess associations between anxiety and depressive symptoms and loneliness (44). These models use variation within individuals to estimate the relationships between variables of interest. Thus, major sources of confounding from all time-invariant confounders that may be correlated with anxiety and depressive symptoms and loneliness (e.g., genetics, sex, race, childhood experiences, lifetime diagnosis of anxiety/depression, etc.) are eliminated (31, 45). Panel models, including reciprocal paths between independent and dependent variables and lagged values of both dependent and independent variables, were used to clarify the direction of the association between anxiety and depressive symptoms and loneliness. The fixed effects term was modeled as a latent variable and allowed to correlate with all time-varying independent variables (46). Allowing these correlations supports the claim that these models control for all time-invariant confounders (31). Cross-lagged association was accommodated by declaring anxiety and depressive symptoms and loneliness as “sequentially exogeneous” independent variables, which allows for the possibility that they could be affected by prior values of the dependent variables. Mechanically, the independent variable at time *t* is allowed to correlate with the error term for the dependent variable at any prior time point (47). COVID-19 containment strategies were not lagged and were included as “strictly exogenous” independent variables, meaning they could not be affected by prior values of the dependent variable. Separate models were created with regression coefficients constrained to be equal or free to vary across time and model fit was compared using the Bayesian information criterion (BIC), a relative fit statistic which approximates the Bayes factor and is typically superior to other fit indices in finding the true model in larger sample sizes (48). A lower BIC indicates a better fitting model, with differences of 0–2, 2–6, 6–10, and >10 indicative of weak, positive, strong, and very strong evidence, respectively (49, 50). Models were also created controlling for social network size and stress as lagged, sequentially exogenous variables. To reduce bias introduced by missing information, full-information maximum likelihood estimation was used (51, 52). Model fits were assessed using the Chi-Square statistic, comparative fit index (CFI), Tucker-Lewis fit index (TLI), and the root mean square error of approximation (RMSEA). Values of CFI and TLI >0.95 and RMSEA values <0.05 are assumed to be indicative of a well-fitting model.

## Results

### Participant Characteristics

Participants who dropped out following the baseline survey had slightly higher depression (*d* = 0.22, 95% CI = 0.14–0.30; *p* < 0.001), anxiety (*d* = 0.11, 0.03–0.19; *p* = 0.011), and loneliness (*d* = 0.15, 0.07–0.24; *p* < 0.001) symptoms compared to the analytic sample. Full baseline characteristics of participants included in the current study are presented in Table 1. Briefly, participants (*n* = 2,361; 75.4% retention rate; 63.9% female) were fairly evenly dispersed across age categories from 18 to 74, with 235 participants aged ≥75 years, and were generally well-educated (88.2% college graduates or above) and overweight (BMI = 26.72 ± 5.69 kg/m^2^). Respondents in the analytic sample were more likely to be female (63.9 vs. 50.8%), white (93.1 vs. 76.3%), and have a higher education level (college graduates 88.2 vs. 31.5%) compared to US adult population data from the 2019 Census Bureau (53).

**Table 1:** Baseline participant characteristics (*n* = 2,361 US adults).

Age (years)	
18–24	318 (13.5)
25–34	354 (15.0)
34–44	316 (13.4)
45–54	300 (12.7)
55–64	397 (16.8)
65–74	441 (18.7)
≥75	235 (10.0)
Sex	
Female	1,508 (63.9)
Male	846 (35.8)
Transgender	7 (0.3)
Race	
White	2,199 (93.1)
Education	
Up to high school graduate	33 (1.4)
Some college	246 (10.4)
Up to college graduate	973 (41.2)
Graduate degree	1,109 (47.0)
Body mass index	26.7 ± 5.7
Smoker (yes)	50 (2.1)
Chronic conditions	
0	131 (5.5)
1	794 (33.6)
≥2	1,435 (60.8)
Not reported	1 (<0.1)
Lifetime diagnosis of depression or anxiety (yes)	592 (25.1)
Public health restrictions	
Self-isolating/quarantining	229 (9.7)
Shelter in place	1,134 (48.0)
None/social distancing	998 (42.3)

*Numbers are N (%) or mean ± standard deviation*.

### Bidirectional Associations Between Anxiety and Depressive Symptoms and Loneliness

**Figure 1:**
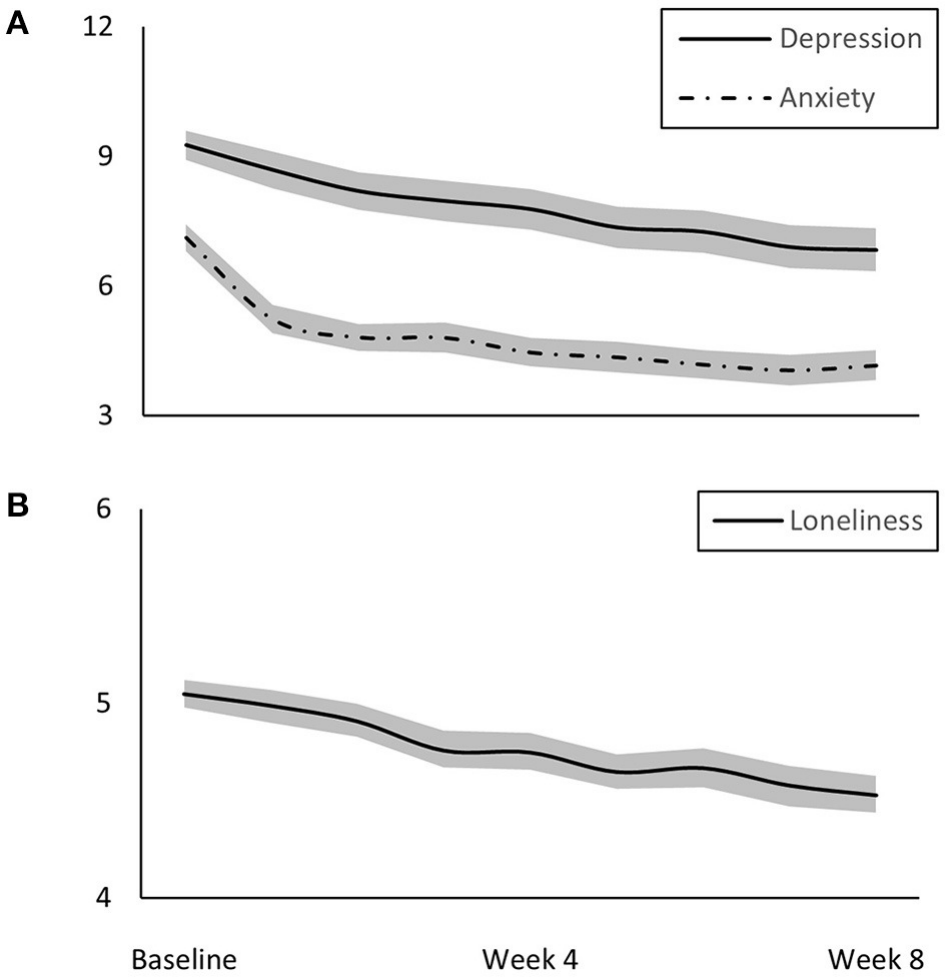
Mean **(A)** depression and anxiety (both range: 0–63) and **(B)** loneliness (range: 3–9) symptom scores with 95% confidence interval bands over 8 weeks of follow-up.

**Figure 2:**
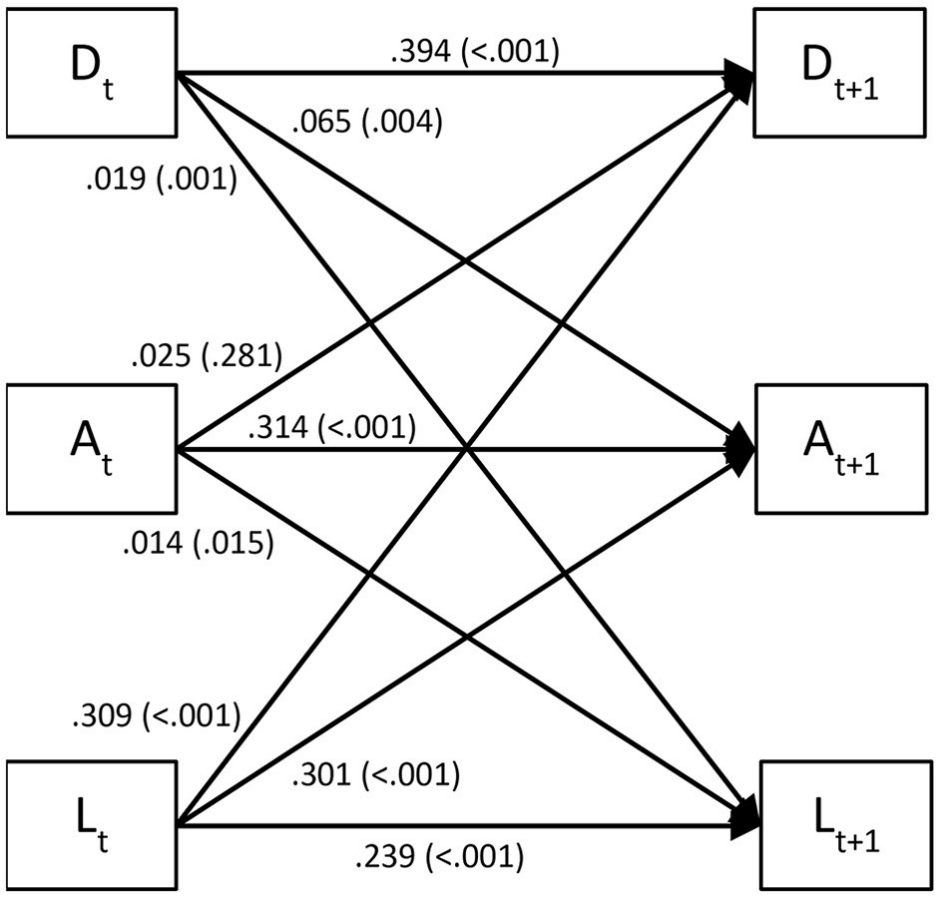
Lagged associations (standardized regression coefficients with *p*-values in parentheses) between depression (D) and anxiety (A) symptoms and loneliness (L) in 2,361 US adults over 8 weeks derived from dynamic panel models with fixed effects. Autoregressive effects are represented as arrows running from a given variable at timepoint *t* to the same variable at timepoint *t*+1. Lagged associations between variables over 1-week intervals (i.e., cross-lagged effects) are illustrated by diagonal arrows.

Mean depressive and anxiety symptom and loneliness scores and their intercorrelations at each time-point are presented in Figure 1 and Supplementary Table 1, respectively. Dynamic panel models with fixed effects were specified to examine reciprocal relationships between anxiety and depressive symptoms and loneliness over 8 weeks. BIC values (Supplementary Table 2) very strongly supported that models with coefficients constrained to be equal across time fit the data better than those with regression coefficients free to vary across time. Model fit statistics (Table 2) indicated that these constrained models fit the data adequately. Figure 2 shows results from the dynamic panel models with fixed effects between anxiety and depressive symptoms and loneliness.

**Table 2:** Fit statistics for the dynamic panel models with fixed and lagged effects between symptoms of depression, anxiety, and loneliness.

**Outcome**	**Chi square**	**df**	**CFI**	**TLI**	**RMSEA (90%CI)**
Depression	523.87	197	0.983	0.975	0.027 (0.024–0.029)
Anxiety	594.62	197	0.975	0.963	0.029 (0.027–0.032)
Loneliness	411.58	197	0.984	0.976	0.021 (0.019–0.024)

*Data were derived from 8 weeks of data in 2,361 US adults*.

*A CFI and TLI ≥ 0.95 and RMSEA < 0.05 are indicative of a well-fitting model*.

*90% CI, 90% confidence interval; CFI, comparative fit index; df, degrees of freedom; RMSEA, Root mean squared error of approximation; TLI, Tucker-Lewis index*.

Depressive symptoms were associated with small subsequent increases in both anxiety symptoms (β = 0.065, 95% CI = 0.022–0.109; *p* = 0.004) and loneliness (β = 0.019, 0.008–0.030; *p* = 0.001). Anxiety symptoms were associated with a small subsequent increase in loneliness (β = 0.014, 0.003–0.025; *p* = 0.015) but not depressive symptoms (β = 0.025, −0.020–0.070; *p* = 0.281). Loneliness was strongly associated with subsequent increases in both depressive (β = 0.309, 0.159–0.459; *p* < 0.001) and anxiety (β = 0.301, 0.165–0.436; *p* < 0.001) symptoms. Compared to social distancing, quarantining or stay-at-home orders were not associated with anxiety or depressive symptoms or loneliness (all *p* ≥ 0.095).

### Are the Associations Between Depressive and Anxiety Symptoms and Loneliness Driven by Perceived Stress and Social Network Size?

The next model examined whether the associations between depressive and anxiety symptoms and loneliness might be attributable to perceived stress and social network size. At baseline, social network size and perceived stress were associated with depressive (social network: *r* = −0.177; *p* < 0.001; stress: *r* = 0.666; *p* < 0.001) and anxiety (social network: *r* = −0.066; *p* = 0.001; stress: *r* = 0.536; *p* < 0.001) symptoms and loneliness (social network: *r* = −0.229; *p* < 0.001; stress: *r* = 0.456; *p* < 0.001). Building on the primary models, social network size and perceived stress were added as sequentially exogenous variables with lagged effects. These models fit the data adequately (Supplementary Table 3).

Results from the dynamic panel models with fixed effects between depressive and anxiety symptoms and loneliness controlling for perceived stress and social network size are presented in Supplementary Figure 1. Perceived stress was associated with a small subsequent increase in depressive symptoms (β = 0.074, 0.004–0.144; *p* = 0.037), but not anxiety symptoms (β = 0.020, −0.040–0.081; *p* = 0.516) or loneliness (β = 0.007, −0.010–0.023; *p* = 0.439). Social network size was associated with a small subsequent decrease in anxiety symptoms (β = −0.153, −0.287-−0.020; *p* = 0.025), but not depressive symptoms (β = −0.058, −0.194−0.079; *p* = 0.409) or loneliness (β = −0.011, −0.045–0.023; *p* = 0.537). Interrelations between depressive and anxiety symptoms and loneliness did not materially differ from primary analyses. Depressive symptoms were associated with small subsequent increases in both anxiety symptoms (β = 0.051, 95% CI = 0.007–0.095; *p* = 0.024) and loneliness (β = 0.017, 0.006–0.029; *p* = 0.003). Anxiety symptoms were associated with a small subsequent increase in loneliness (β = 0.014, 0.003–0.025; *p* = 0.013) but not depressive symptoms (β = 0.027, −0.018–0.073; *p* = 0.239). Loneliness was strongly associated with subsequent increases in both depressive (β = 0.272, 0.124–0.421; *p* < 0.001) and anxiety (β = 0.285, 0.152–0.419; *p* < 0.001) symptoms.

## Discussion

This study examined bidirectional relationships between depressive and anxiety symptoms and loneliness in 2,361 US adults over 8 weeks during the COVID-19 pandemic. Findings showed that (1) loneliness was strongly associated with subsequent increases in depressive and anxiety symptoms, but anxiety and depressive symptoms were only weakly associated with subsequent increases in loneliness; (2) depressive symptoms were associated with subsequent increases in anxiety symptoms, but not vice versa; and, (3) COVID-19 containment strategies were not associated with depressive or anxiety symptoms or loneliness. These findings are independent of time-invariant factors (e.g., genetics, sex, race, adverse childhood experiences, etc.), reverse causation, and time-varying COVID-19 containment strategies, social network size, and perceived stress.

Bidirectional associations between loneliness and depressive/anxiety symptoms were observed, although loneliness was a considerably stronger predictor of depressive and anxiety symptoms relative to the reverse causal direction. Previous evidence for associations between loneliness and depressive symptoms has been mixed and is scarce for loneliness and anxiety symptoms. Findings from the Chicago Health, Aging, and Social Relations Study indicated that loneliness predicted subsequent changes in depressive symptomatology but not vice versa (24). However, most research has demonstrated bidirectional relationships, albeit with variability in the magnitudes of the associations (14, 22, 23). In contrast to the current study, these previous studies have focused on middle-aged to older adult cohorts and had follow-up periods ranging from 2 to 14 years. It seems plausible that age and follow-up period may influence the relationships of interest, although future research is required to test if and how they do moderate the loneliness-mental health relationships. Additionally, compared to these prior studies, an important strength of the current study was the use of standard fixed effect methods which effectively rule out all time-invariant confounding, a central threat to valid causal inference in epidemiological studies, which may contribute to some differences in findings.

Over the course of the 8-week follow-up, anxiety and depressive symptoms and loneliness decreased by ~10% each, potentially as people adjusted to their “new normal.” This is encouraging as it may suggest that the initial mental health impact of the pandemic may not persist. However, there may still be longer-term effects, particularly relative to loneliness which can increase due to economically and socially challenging conditions (29). As increases in loneliness were strongly associated with increases in depressive and anxiety symptoms, addressing this may be a key factor in the maintenance of mental health as the pandemic and its subsequent impact progress.

Meta-analytic evidence has shown that depressive and anxiety symptoms predict one another with moderate and similar strength (*r* = 0.31–0.34), with relationships stronger over shorter time periods and weaker over longer time periods (54). Of the 35 studies included in these analyses, just one had a comparable follow-up period to the current study, but it focused on anxiety patients rather than the general population. This difference in follow-up period may explain why the magnitude of the association for depressive symptoms predicting anxiety symptoms in the current study is smaller than that found in the prior meta-analysis and why anxiety symptoms did not predict depressive symptoms.

Throughout the COVID-19 pandemic, considerable concern has been expressed about the potential mental health impacts of the various containment strategies [e.g., social distancing, stay-at-home orders, etc.; (55)]. Longitudinal data from the UK demonstrated that mental health had deteriorated compared with pre-COVID-19 trends (7). In this study, being young, a woman, and living with children, especially preschool-age children, were strongly associated with increases in mental distress. However, the entire sample was under strict lockdown and adherence was not assessed, so associations between different recommended or actual containment behaviors and mental health were not examined. Previous cross-sectional evidence from the current cohort showed that, compared to individuals who were social distancing, individuals who were self-isolating reported higher depressive and anxiety symptoms (35). However, the present longitudinal findings showed no associations between containment strategies and depressive and anxiety symptoms or loneliness. This suggests that factors external to the containment strategy to which a person is adhering underpin the previously observed deteriorations in mental health.

### Limitations

Several potential limitations of this study should be noted. Firstly, although the use of fixed effects models effectively rule out time-invariant confounding, and reverse causation is controlled for by alternatively using panel models allowing for lagged and reciprocal relationships, experimental evidence would confer greater confidence in the causal role of loneliness on depressive and anxiety symptoms and vice versa. Secondly, the use of a convenience sample resulted in a sample that was not representative of the US population, thereby limiting the generalisability of the findings. Additionally, participants who dropped out following the baseline survey had slightly higher depressive and anxiety symptoms and loneliness, meaning the analytic sample had comparatively better mental health. This could lead to an underestimation of the true effect as a potential negative feedback loop between depressive and anxiety symptoms may be stronger among those with worse mental health. Thirdly, self-reported depressive and anxiety symptom measures could lead to more measurement error than clinical interviews, though such measurement error was not expected to be related to our hypotheses. Nonetheless, whether the current findings for depressive and anxiety symptoms extend to clinical diagnoses is unknown. Finally, future research is required to test whether the relationships observed here persist beyond the pandemic.

## Conclusions

These findings contribute to growing evidence that supports the longitudinal bidirectional depressive symptom–loneliness relationship, and provide novel evidence for a bidirectional anxiety symptom–loneliness relationship; however, loneliness was a stronger predictor of depressive and anxiety symptoms relative to the reverse causal direction. High loneliness may be a key risk factor for the development of future anxiety or depressive symptoms, underscoring the need to combat or prevent loneliness both throughout and beyond the COVID-19 pandemic.

## Data Availability Statement

The raw data supporting the conclusions of this article will be made available by the authors, without undue reservation.

## Ethics Statement

The studies involving human participants were reviewed and approved by Iowa State University's Institutional Review Board. The patients/participants provided their written informed consent to participate in this study.

## Author Contributions

CM: analysis and interpretation of data and drafting of the manuscript. All authors study concept and design and revision of the manuscript.

## Funding

CM was funded by the Irish Research Council under the Government of Ireland Postdoctoral Programme.

## Conflict of Interest

The authors declare that the research was conducted in the absence of any commercial or financial relationships that could be construed as a potential conflict of interest.

## Publisher's Note

All claims expressed in this article are solely those of the authors and do not necessarily represent those of their affiliated organizations, or those of the publisher, the editors and the reviewers. Any product that may be evaluated in this article, or claim that may be made by its manufacturer, is not guaranteed or endorsed by the publisher.
