# Supplementary material for: Bidirectional Associations Between Depressive and Anxiety Symptoms and Loneliness During the COVID-19 Pandemic: Dynamic Panel Models With Fixed Effects
Source: Front Psychiatry. 2021 Dec 9;12:738892. doi: 10.3389/fpsyt.2021.738892 (PMC8695764; doi:10.3389/fpsyt.2021.738892)
Supplement: Supplementary file 1 [file Data_Sheet_1.docx]

**Supplementary Table 1**. Intercorrelations between depression (bdi_total) and anxiety (bai_total) symptoms and loneliness (lone_total) at each assessment

| \| **Supplementary Table 2**. Number of observations, means, and standard deviations for loneliness, depressive symptoms, anxiety symptoms, social network size, and stress at each timepoint \| \| \| \| \| \| \| \| \| \| \| \| --- \| --- \| --- \| --- \| --- \| --- \| --- \| --- \| --- \| --- \| --- \| \|  \|  \| T1 \| T2 \| T3 \| T4 \| T5 \| T6 \| T7 \| T8 \| T9 \| \| Loneliness \| Observations \| 2360 \| 1614 \| 1500 \| 1365 \| 1342 \| 1275 \| 1196 \| 1128 \| 1166 \| \| Mean \| 5.1 \| 5.0 \| 4.9 \| 4.8 \| 4.8 \| 4.7 \| 4.7 \| 4.6 \| 4.5 \| \| SD \| 1.8 \| 1.7 \| 1.7 \| 1.7 \| 1.7 \| 1.7 \| 1.7 \| 1.8 \| 1.7 \| \| Depressive symptoms \| Observations \| 2361 \| 1615 \| 1501 \| 1365 \| 1342 \| 1275 \| 1196 \| 1128 \| 1167 \| \| Mean \| 9.3 \| 8.7 \| 8.2 \| 8.0 \| 7.8 \| 7.4 \| 7.3 \| 6.9 \| 6.8 \| \| SD \| 8.3 \| 8.7 \| 8.5 \| 8.9 \| 8.7 \| 8.7 \| 8.5 \| 8.6 \| 8.8 \| \| Anxiety symptoms \| Observations \| 2361 \| 1615 \| 1501 \| 1365 \| 1342 \| 1275 \| 1196 \| 1128 \| 1167 \| \| Mean \| 7.1 \| 5.2 \| 4.8 \| 4.8 \| 4.5 \| 4.4 \| 4.2 \| 4.1 \| 4.2 \| \| SD \| 7.8 \| 6.6 \| 6.2 \| 6.5 \| 6.1 \| 6.4 \| 5.7 \| 6.0 \| 6.1 \| \| Social network size \| Observations \| 2361 \| 1615 \| 1501 \| 1365 \| 1342 \| 1275 \| 1196 \| 1128 \| 1167 \| \| Mean \| 8.5 \| 8.3 \| 8.4 \| 8.4 \| 8.5 \| 8.6 \| 8.7 \| 8.7 \| 8.7 \| \| SD \| 2.6 \| 2.7 \| 2.7 \| 2.8 \| 2.8 \| 2.7 \| 2.7 \| 2.8 \| 2.8 \| \| Stress \| Observations \| 2358 \| 1612 \| 1500 \| 1365 \| 1342 \| 1275 \| 1196 \| 1128 \| 1166 \| \| Mean \| 6.0 \| 5.2 \| 4.9 \| 4.8 \| 4.7 \| 4.4 \| 4.3 \| 4.0 \| 4.2 \| \| SD \| 3.0 \| 3.1 \| 3.1 \| 3.2 \| 3.3 \| 3.2 \| 3.3 \| 3.2 \| 3.3 \| \| SD=standard deviation \| \| \| \| \| \| \| \| \| \| \|   **Supplementary Table 3**. BIC criterion for primary models where the cross-lagged regression coefficients were constrained to be equal over time and for models where parameters varied freely | | |
| --- | --- | --- | --- | --- | --- | --- | --- | --- | --- | --- | --- | --- | --- | --- | --- | --- | --- | --- | --- | --- | --- | --- | --- | --- | --- | --- | --- | --- | --- | --- | --- | --- | --- | --- | --- | --- | --- | --- | --- | --- | --- | --- | --- | --- | --- | --- | --- | --- | --- | --- | --- | --- | --- | --- | --- | --- | --- | --- | --- | --- | --- | --- | --- | --- | --- | --- | --- | --- | --- | --- | --- | --- | --- | --- | --- | --- | --- | --- | --- | --- | --- | --- | --- | --- | --- | --- | --- | --- | --- | --- | --- | --- | --- | --- | --- | --- | --- | --- | --- | --- | --- | --- | --- | --- | --- | --- | --- | --- | --- | --- | --- | --- | --- | --- | --- | --- | --- | --- | --- | --- | --- | --- | --- | --- | --- | --- | --- | --- | --- | --- | --- | --- | --- | --- | --- | --- | --- | --- | --- | --- | --- | --- | --- | --- | --- | --- | --- | --- | --- | --- | --- | --- | --- | --- | --- | --- | --- | --- | --- | --- | --- | --- | --- | --- | --- | --- | --- | --- | --- | --- | --- | --- | --- | --- | --- | --- | --- | --- | --- | --- | --- | --- | --- | --- | --- | --- | --- | --- | --- | --- |
| **Outcome** | **Constrained parameters BIC** | **Free parameters BIC** |
| Depression | 164501.8 | 164669.2 |
| Anxiety | 163533.1 | 163713.1 |
| Loneliness | 151925.4 | 152105.2 |
| Note: A lower BIC is indicative of a better fitting model.  Abbreviations: BIC=Bayesian information criterion | | |

| **Supplementary Table 4**. Fit statistics for the dynamic panel models with fixed and lagged effects between symptoms of depression, anxiety, and loneliness, controlling for social support and stress. Data were derived from eight weeks of data in 2361 US adults. | | | | | |
| --- | --- | --- | --- | --- | --- |
| **Outcome** | **Chi square** | **df** | **CFI** | **TLI** | **RMSEA (90%CI)** |
| Depression | 602.52 | 251 | 0.982 | 0.971 | 0.024 (0.022–0.027) |
| Anxiety | 642.55 | 251 | 0.975 | 0.959 | 0.026 (0.023–0.028) |
| Loneliness | 470.63 | 251 | 0.984 | 0.973 | 0.019 (0.017–0.022) |
| Note: A CFI and TLI ≥0.95 and RMSEA <0.05 are indicative of a well-fitting model.  Abbreviations: 90%CI=90% confidence interval; CFI=comparative fit index; df=degrees of freedom; RMSEA=Root mean squared error of approximation; TLI=Tucker-Lewis index | | | | | |


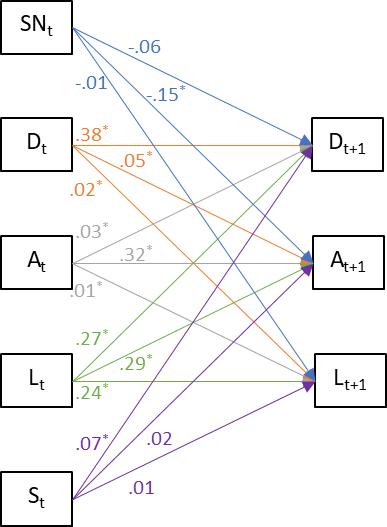


**Supplementary Figure 1**. Lagged associations (standardised regression coefficients) between social network (SN), depression (D), anxiety (A), loneliness (L), and stress (S) in 2361 US adults throughout eight weeks of the COVID-19 pandemic derived from dynamic panel models with fixed effects. *indicates significance at the p<0.05 level
